# Peroxisome proliferator-activated receptor activating hypoglycemic effect of *Gardenia jasminoides* Ellis aqueous extract and improvement of insulin sensitivity in steroid induced insulin resistant rats

**DOI:** 10.1186/1472-6882-14-30

**Published:** 2014-01-18

**Authors:** Ying-I Chen, Yu-Wen Cheng, Chung-Yuh Tzeng, Yu-Chen Lee, Yaw-Nan Chang, Shih-Chieh Lee, Chin-Chun Tsai, Jaw-Chyun Chen, Jason Tze-Cheng Tzen, Shih-Liang Chang

**Affiliations:** 1 Graduate Institute of Biotechnology, National Chung Hsing University, Taichung, Taiwan; 2 College of Life Sciences, National Tsing Hua University, Hsinchu, Taiwan; 3 Department of Internal Medicine, Lee’s General Hospital, Miaoli, Taiwan; 4 Department of Orthopedics, Taichung Veterans General Hospital, Taichung City, Taiwan; 5 Department of Acupuncture, China Medical University Hospital and School of Chinese Medicine, China Medical University, Taichung, Taiwan; 6 Department of Biotechnology, National Formosa University, Yunlin County, Taiwan; 7 Department of BioIndustry technology, Da-Yeh University, Changhua County, Taiwan; 8 Chinese Medicine Department E-D Hospital, I-Shou University, Kaohsiung, Taiwan; 9 School of Chinese Medicine for Post-Baccalaureate, I-Shou University, Kaohsiung, Taiwan; 10 Department of Medicinal Botanicals and Health Applications, Da-Yeh University, Changhua, Taiwan

**Keywords:** Gardenia, Blood glucose, Insulin resistance, Peroxisome proliferator-activated receptor

## Abstract

**Background:**

The active components of Gardenia (*Gardenia jasminoides* Ellis, GJ) exhibit a hypoglycemic effect by improving insulin secretion and lowering plasma lipids. In the present study, we fed a water extract of gardenia to steroid-induced insulin-resistant (SIIR) rats and observed changes in signaling proteins in order to elucidate the mechanisms of the insulin-sensitizing effect of GJ and evaluate its possibility as an insulin-sensitizing agent.

**Methods:**

Normal Wistar rats were randomly divided into a control group (i.e., saline) and experimental groups (GJ 100 and 200 mg/kg). Blood samples were taken at 0, 30, and 60 min for plasma glucose assay in order to determine the optimal dose to induce the hypoglycemic effect. SIIR rats were then randomly divided into a control group (i.e., saline) and an experimental group (optimal dose of gardenia extract) to observe the insulin-sensitizing effect of the extract. Finally, western blot analysis was performed to detect intracellular signaling proteins to elucidate the mechanisms of the insulin-sensitization effect of GJ.

**Results:**

The normal Wistar rats in the GJ 200 mg/kg group exhibited significant hypoglycemic activity. Meanwhile, the SIIR rats had higher plasma glucose levels than normal rats. There was no obvious change in insulin level, but the insulin sensitivity index and homeostasis model assessment index were significantly elevated. Meanwhile, a significant hypoglycemic effect was observed with GJ 200 mg/kg. In addition, intracellular signaling proteins including insulin receptor substrate-1 (IRS-1) and peroxisome proliferator-activated receptor (PPARγ) were elevated in muscle cells.

**Conclusions:**

The optimal dose of GJ aqueous extract of 200 mg/kg exerts a PPARγ-activating hypoglycemic effect and improves insulin resistance in SIIR rats. Therefore, it is a potential insulin-sensitizing agent in type 2 diabetes mellitus with insulin resistance.

## Background

Good control of blood glucose can effectively reduce the microvascular and macrovascular complications of diabetes mellitus (DM). Lifestyle modifications such as deceasing body weight, exercise, and diet control can decrease insulin resistance (IR). In turn, this can improve plasma glucose level and decrease the HbA1c level to 1% to 2% [1]. Unfortunately, most patients with DM only transiently maintain stable plasma glucose levels and ultimately require medication therapy.

The main targets of insulin are the muscle, liver, and adipose tissue; therefore, IR [2] induces abnormal glucose and lipid metabolism [3]. Several factors can induce IR, including decreased physical activity, high-fat diet, medications, and glucose intoxication. Some studies indicate that increased serum free fatty acid induces IR as well as obesity and type 2 DM [4,5].

IR and inadequate insulin secretion are the main mechanisms of type 2 DM, but IR may manifest 5 to 10 years prior to DM development. Metabolic syndrome, which comprises IR, obesity, hyperlipidemia, and hypertension [6,7], can increase the risk of cardiovascular disease by 79%, especially large-vessel disease and DM [8,9]. According to the diagnostic criteria of the National Cholesterol Education Program Expert Panel, Adult Treatment Panel III, the incidence of metabolic syndrome is 23.9% [10]. There are several ways of improving IR, including diet modification, exercise, medication, acupuncture, and massage. Moreover, there are several categories of oral anti-diabetic agents, including α-glycosidase inhibitors, biguanides, sulfonylureas, benzoic acid derivatives, phenylalanines, and thiazolidinediones [11-16]. Thiazolidinediones are specific anti-diabetic agents because they can modify genes regulating lipids and carbohydrates by activating peroxisome proliferator-activated receptors (PPARs) [17,18].

*Gardenia jasminoides* (GJ) has the potential to induce hypoglycemia and decrease serum lipid concentrations. One study reports that geniposide derived from GJ inhibits glucose phosphate and glucose-6-phosphatase activity, decreasing glycogenolysis and glucose release into the blood [19]. Ursolic acid, a component of GJ, can prevent diabetic complications by improving lipid metabolism and polyol pathways as well as inhibiting glycogenolysis. The active compounds of GJ are crocin [20], geniposide, and genipin. Several studies have confirmed that GJ protects liver function and has anti-inflammatory, hypoglycemic, and lipid-lowering effects [21].

IR usually progresses to DM over the course of several years [22]. Therefore, if IR can be successfully treated, DM should be preventable [23]. Thiazolidinediones are a type of oral anti-diabetes agent administered to improve IR. The first commercially available medicine in this category was troglitazone (Rezulin®), which was introduced in 1997 [24,25]. Some prospective studies confirmed that this drug decreases serum insulin level and preserves the insulin secretion function of islet cells. In addition, it can even prevent type 2 DM development [26,27]. However, it induces hepatotoxicity, which resulted in hepatic failure in some patients with diabetes, necessitating liver transplantation or even causing mortality; therefore, it was ultimately withdrawn from the market [28-30]. Rosiglitazone (Avandia®) and pioglitazone (Actos®) are the only thiazolidinediones currently available; their common hypoglycemic mechanism is the activation of PPARγ [31,32].

Our previous studies revealed that GJ has a hypoglycemic effect. In normal Wistar rats, GJ effectively induces insulin secretion and reduces blood glucose levels, simultaneously eliciting increases in insulin receptor substrate-1 (IRS-1) and PPARγ signals. Therefore, cholinergic nerve activation is involved in the hypoglycemic mechanism of GJ. Rats with dexamethasone-induced type 2 diabetes do not have sufficient insulin. Therefore, the present study investigated whether aqueous extracts of GJ produce such hypoglycemic effects in rats with diabetes.

In order to evaluate the effects of hypoglycemia and improved IR, we administered GJ aqueous extracts to steroid-induced insulin-resistant (SIIR) rats. In addition, western blotting was used to evaluate intracellular signaling proteins to study the possible underlying mechanisms [33].

## Methods

### Animal model

Normal male Wistar rats weighing approximately 250–350 g and aged 8–10 weeks were purchased from the BioLASCO Animal Center, Taipei, Taiwan. SIIR rats were created by administrating dexamethasone (1 mg⋅kg^-1^⋅day^-1^ i.p. for 5 days) via the femoral vein in a fasting state [34,35]. Animals were housed in Plexiglas cages held at a room temperature of 25 *±* 2°C with a relative humidity of 60 *±* 5%. Rats were fed standard rat chow and water *ad libitum*. Animals were randomly divided into experimental and control groups after a 1-week acclimation period. All animals were anesthetized using pentobarbital (40 mg/kg i.p.). All animals were treated in accordance with the National Institute of Health Guide for the Care and Use of Laboratory Animals, and the study protocol was approved by the ethics committee of the Da-Yeh University, Changhua, Taiwan.

### GJ aqueous extract

GJ Ellis extract was provided by Challenge Bioproducts Co., Ltd. (Taiwan). The powder was mixed with normal saline to create a solution (500 mg/mL), which was stored at 4°C.

### Plasma glucose assay

Animals were anesthetized using pentobarbital (40 mg/kg i.p.). Approximately 0.3–0.5 mL blood was obtained from a femoral vein using a 1-mL syringe containing heparin. Blood was obtained for glucose assay 30 min after treatment (baseline, 0 min) and 30 and 60 min after baseline. The collected blood was placed into Eppendorf tubes, shaken lightly, and stored on ice. Following centrifugation at 21,880 × *g* for 5 min, Glucose UV reagent (Raichem, USA) was added to assay the amount of biological index glucose contained in the serum. The content was measured using a fully automatic biochemical analyzer (Roche COBAS-MIRA-PLUS, USA). Hypoglycemic activity (%) was calculated as follows: [(glucose level at 30 or 60 min - glucose level at baseline)/glucose level at baseline] × 100%.

### Hypoglycemic effect measurement

#### Optimal dose of GJ for the hypoglycemic effect

Normal Wistar rats were separated randomly into 2 experimental groups: GJ 100 (oral feeding GJ, 100 mg/kg) and GJ 200 (oral feeding GJ, 200 mg/kg), and a control group (oral feeding normal saline). Each group consisted of 6 rats.

#### Comparisons between normal Wistar and SIIR rats

Normal Wistar rats were separated randomly into saline and SIIR groups (*n =* 6 each). The rats in the SIIR group were induced into SIIR by dexamethasone as described above. The same procedure was performed in the saline group, except saline was injected instead. Blood samples were taken at 0, 30, and 60 min.

#### Hypoglycemic activity of GJ in SIIR rats

The SIIR rats were divided into 2 groups (*n =* 6 each). The baseline (i.e., 0 min) glucose level was checked 30 min after solution feeding. Another blood glucose sample was drawn 30 min after the baseline (i.e., 30 min).

### Insulin and insulin resistance assay

The concentration of plasma insulin was analyzed by human enzyme-linked immunosorbent assay (Seminariegatan 29, S-752 28, Mercodia AB, Uppsala, Sweden) in both groups. IR was assessed according to the homeostasis model assessment index calculated using the following formula: (fasting plasma insulin level [μU/mL] × fasting plasma glucose [mmol/L])/22.5 [36]; Insulin sensitivity is expressed as insulin sensitivity index (ISI), which was calculated as follows: ISI = 1/(log[fasting insulin μU/mL] + log[fasting glucose mg/dL]) [37]. The results of this experiment are expressed as ISI × 10^3^.

### Western blotting

At the end of treatment (i.e., 60 min) in each group, portions of the gastrocnemius muscles were taken as samples to analyze the insulin signaling proteins, IRS-1 and PPARγ. Muscle samples were homogenized in buffer solution before centrifugation at 21,880 × *g*. The supernatants were used to estimate the amount of protein using an assay kit from Bio-Rad Laboratories. The supernatant (i.e., protein) was mixed with 4× sodium dodecyl sulfate loading dye and boiled for 15 min at 95°C for denaturation. Separating (8%) and stacking gels were prepared. Protein in buffer (90 μg/mL) was subsequently loaded into each well for electrophoresis. Proteins were electrophoretically transferred to polyvinylidene difluoride membranes at 4°C. The membranes were then blocked with 5% nonfat dry milk in phosphate-buffered saline for 1 h at room temperature and incubated with specific primary antibodies (Santa Cruz Biotechnology, Inc.). After the membranes were washed in buffer containing 0.1% Tween 20 in 1*×* phosphate-buffered saline, the blots were incubated with horseradish peroxidase-linked specific secondary antibody (Santa Cruz Biotechnology, Inc.) and evaluated using an enhanced chemiluminescence detection using ECL Reagent Plus (PerkinElmer Life Sciences, Inc.). Band intensities were quantified by densitometry to observe the target proteins.

### Statistical analysis

The experimental results of all groups are expressed as mean *±* standard error of mean; Student’s *t*-test and analysis of variance were performed to determine significance. The level of statistical significance was set at *P* < 0.05.

## Results

### Optimal dose of GJ for hypoglycemia

In the normal Wistar rats, plasma glucose levels were checked at 0, 30, and 60 min to determine the hypoglycemic effect in the control and 2 experimental groups (i.e., GJ and GJ 200). The GJ-fed groups exhibited a tendency towards decreasing glucose level with time. The GJ 200 group exhibited a significantly greater hypoglycemic effect than the control and GJ 100 groups.

**Figure 1:**
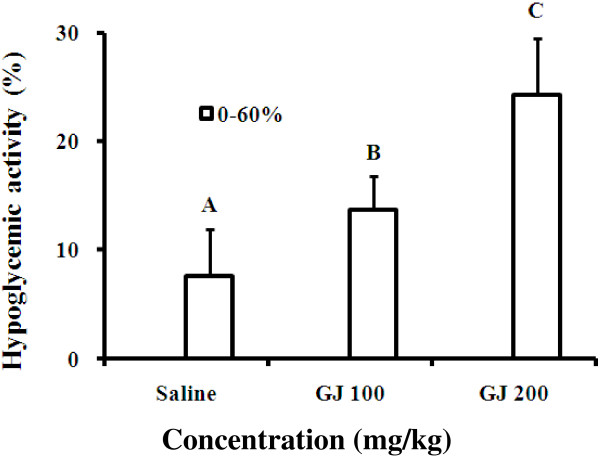
**Hypoglycemic activity of each group.** Gardenia jasminoides (GJ) 100: feeding GJ 100 mg/kg; GJ 200: feeding GJ 200 mg/kg; saline: feeding normal saline. p < 0.05 vs. saline, A < B < C by analysis of variance, post hoc: least significant difference test, n = 6.

In the 2 experimental groups, the plasma glucose level of the GJ 100 group did not change significantly until 60 min. Furthermore, the GJ 200 group exhibited significantly decreased plasma glucose levels starting at 30 min. Therefore, we used GJ 200 mg/kg as the therapeutic dose in subsequent experiments (Table 1). In addition, the hypoglycemic activities (%) changed in a dose-dependent manner. The GJ 200 group exhibited the best hypoglycemic activity (Figure 1).

**Table 1:** Plasma glucose changes by group

**Group**	**Plasma glucose (mg/dL)**		
	**0 min**	**30 min**	**60 min**
Saline	113.45 ± 13.74	108.28 ± 11.01	103.35 ± 11.62
GJ 100	117.46 ± 6.84	110.05 ± 7.69	101.28 ± 5.02^*^
GJ 200	127.86 ± 7.74	108.65 ± 7.95^*^	99.62 ± 9.52^**^

GJ: *Gardenia jasminoides*; GJ 100: feeding GJ 100 mg/kg; GJ 200: feeding GJ 200 mg/kg; saline: feeding normal saline. *p < 0.05, **p < 0.001 vs. plasma glucose at 0 min, Student’s *t*-test, *n* = 6.

### Hypoglycemic effect of GJ in SIIR rats

**Figure 2:**
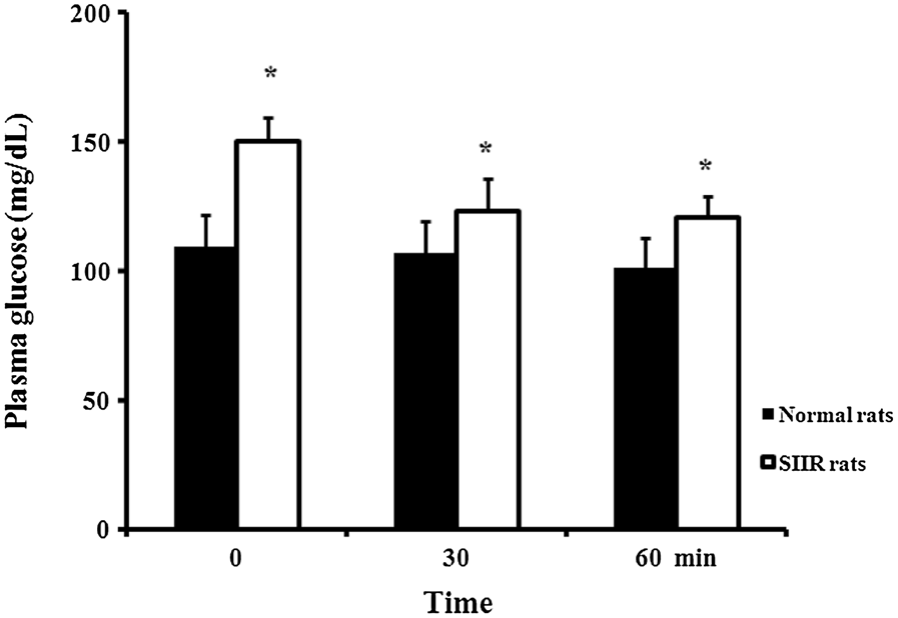
**Plasma glucose levels of steroid-induced insulin-resistant (SIIR) rats vs. normal Wistar rats.** * p < 0.05 vs. 0 min, Student’s t-test, n = 6.

**Figure 3:**
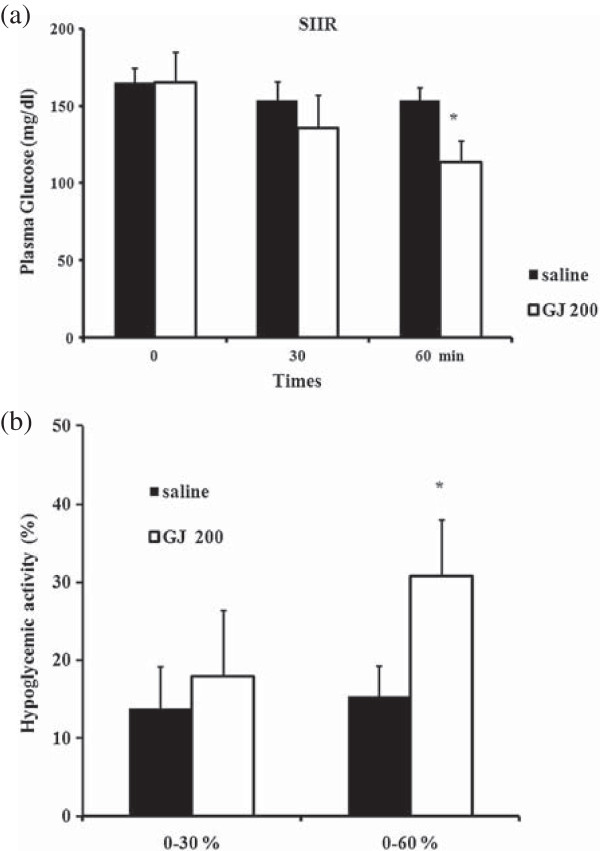
**The hypoglycemic effect of GJ in SIIR rats. (a)** Plasma glucose level and **(b)** hypoglycemic activity in the saline and Gardenia jasminoides (GJ) 200 steroid-induced insulin-resistant (SIIR) rats. * p < 0.05 vs. saline, Student’s t-test, n = 6.

The SIIR rats exhibited higher serum glucose levels than those of the saline group (Figure 2). The serum glucose levels of SIIR rats fed saline at 0, 30, and 60 min were 165.1, 153.3, and 153.7 mg/dL, respectively, compared to 165.1, 135.5, and 113.6 mg/dL in the GJ 200 group of SIIR rats, respectively. There was a significant difference between the 2 experimental groups at 60 min (Figure 3a). In addition, the hypoglycemic activity in the GJ 200 group was 17.9% and 30.8% at 30 and 60 min, respectively; both results are significantly different from those of the saline group at the corresponding times (Figure 3b).

### Improved insulin sensitivity of SIIR rats after GJ administration

The SIIR rats treated with GJ 200 mg/kg exhibited a significant hypoglycemic effect at 30 and 60 min with no obvious change in insulin level (from 178.92 to 165.77 mU/L) (Table 2). The ISI increased significantly (from 0.67 to 1.05), while the homeostasis model assessment index decreased significantly (from 7.23 to 3.57) (Table 2).

**Table 2:** Changes in insulin sensitivity after GJ 200 feeding to SIIR rats

		**0 min**	**30 min**	**60 min**
SIIR GJ 200	Glucose (mg/dL)	150.54 ± 12.84	125.42 ± 7.59^*^	105.90 ± 0.61^**^
(mg/kg)	Insulin (μU/mL)	178.92 ± 17.30	170.07 ± 17.41	165.77 ± 25.63
	ISI × 10^3^	0.67 ± 0.01	0.85 ± 0.06^*^	1.05 ± 0.18^*^
	HOMA	7.23 ± 2.0	3.76 ± 0.72^*^	3.57 ± 0.45^*^

GJ: *Gardenia jasminoides*; SIIR: steroid induced insulin resistant; ISI: insulin sensitivity index = 1/(log[fasting insulin μU/mL] + log[fasting glucose mg/dL]). Results are expressed as ISI × 10^3^ in this experiment; HOMA: homeostasis model assessment = (fasting plasma insulin level [μU/mL] × fasting plasma glucose [mmol/L])/22.5. *p < 0.05, **p < 0.005 vs. 0 min, Student’s *t*-test, *n* = 6.

### Changes in intracellular signaling proteins in SIIR rats after GJ feeding

**Figure 4:**
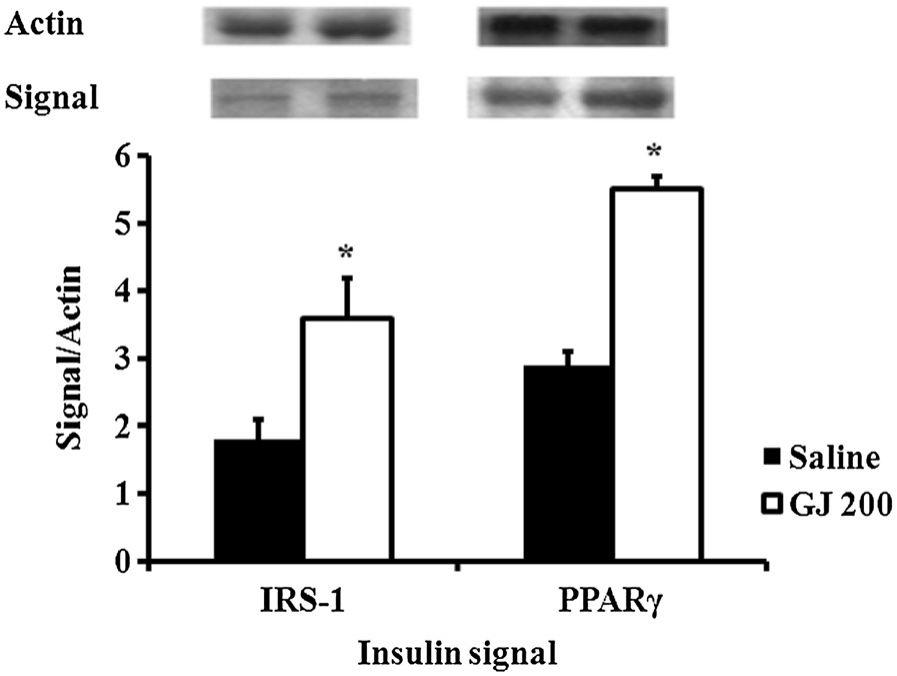
**Change in intracellular insulin signaling protein to actin ratio in steroid-induced insulin-resistant (SIIR) rats 60 min after treatment.** IRS-1: insulin receptor substrate-1, PPAR: peroxisome proliferative-activated receptor. p < 0.05 vs. saline, Student’s t-test, n = 6.

Sixty min after feeding GJ 200 mg/kg to the SIIR rats, the levels of the intracellular signaling proteins, IRS-1 and PPARγ, in the skeletal muscular cell were elevated (Figure 4).

## Discussion

The GJ doses of 100 and 200 mg/kg were chosen on the basis of the study of Kim et al. [33]. GJ 100 mg/kg had a hypoglycemic effect at 60 min. However, a significant decrease in plasma glucose levels was observed 30 min after GJ 200 mg/kg feeding. Thus, the hypoglycemic effect of GJ 200 mg/kg was more obvious at 60 min. Therefore, we used the more effective dose, GJ 200 mg/kg, in subsequent experiments (Table 1 and Figures 1 and 2).

Dexamethasone is a type of adrenal hormone that has an anti-inflammatory effect. However, it also causes patients with DM to require additional insulin doses and increases IR [38]. In the present study, rats were injected with dexamethasone to induce IR. Dexamethasone can induce gluconeogenesis and increase plasma glucose levels and inhibit insulin secretion. In an animal experiment, steroid therapy decreased the expression of glucose transporter-2 protein in β cells [39,40]. Dexamethasone also decreases adenosine monophosphate levels [41] and activates voltage-gated potassium channels [42]. A study reported increased β-cell apoptosis after corticosteroid receptor activation [38]. Therefore, insulin secretion is inhibited by dexamethasone. These mechanisms are associated with exacerbated DM control and its complications after corticosteroid therapy.

GJ extracts lowered plasma glucose level in the SIIR rats. Several previous studies report that GJ has a hypolipidemic effect. One study reported that lower blood lipid levels are associated with decreased plasma glucose levels [19]. This hypoglycemic mechanism may be due to the improvement of IR or enhancement of signaling proteins in the insulin signaling pathways [19].

A study reports that long-term oral administration of GJ results in a hypolipidemic effect. In other studies, Obesity animals were fed GJ, which exerted a hypolipidemic effect; meanwhile, GJ was fed for 1 to 4 weeks before serum lipid examination in other studies [8]. Since the hypolipidemic effect is beneficial for the action of insulin to lower plasma glucose levels, this is possibly why insulin sensitivity was elevated in the present study. Because of the limitation of this study’s design, the 60-min experimental duration was too short to fully observe changes in plasma lipids (unpublished results). The long-term effect of GJ on the relationship between plasma lipids and IR is worth investigating in the future.

IR develops mainly in the liver, skeletal muscle, and adipose tissue. Skeletal muscle cells have difficulty converting plasma glucose into glycogen under IR conditions [43]. In the liver, insulin reduces glycogenolysis and gluconeogenesis [3]. Therefore, the energy of food can be stored, keeping plasma glucose within the normal range. However, when IR develops, such homeostasis is blocked and hyperglycemia is not preventable [44]. In the experiment analyzing changes in intracellular insulin signaling proteins in SIIR rats, PPARγ signaling was augmented after GJ feeding (Figure 4). This hypoglycemic mechanism is similar to that observed with thiazolidinediones.

The main hypoglycemic mechanism of thiazolidinediones occurs in adipose tissue, enhancing free fatty acid uptake by adipocytes [45]. Thiazolidinediones can also lower serum triglyceride and non-esterified fatty acids. PPARγ activation also induces adipocyte differentiation and decreases glucose release from the liver, enhancing glycogen storage in skeletal muscle cells [40]. Since the hypoglycemic effect of GJ is similar to that of thiazolidinediones, GJ may be able to induce insulin sensitivity and prevent DM development. Thus, GJ has the potential to be used as an insulin sensitizer and even as a substitute for thiazolidinediones [19].

## Conclusions

In conclusion, improvement of IR can effectively lower plasma glucose and prevent hyperinsulinemia and/or lower the required doses of oral anti-diabetes agents. Thiazolidinediones effectively decrease IR, but their side effects limit their clinical application [28-30]. Although the hypoglycemic effect of GJ is similar to that of thiazolidinediones, it is possible that GJ will not cause similar side effects in clinical therapy. Therefore, GJ is a potential insulin-sensitizing agent for patients with type 2 DM and/or adjuvant therapy [19,20].

## Abbreviations

DM: Diabetes mellitus; GJ: *Gardenia jasminoides*; ISI: Insulin sensitivity index; PPAR: Peroxisome proliferator-activated receptor; SIIR: Steroid-induced insulin-resistant.

## Competing interests

The authors declare that they have no competing interests.

## Authors’ contributions

YIC, YWC and JCC drafted the manuscript, and acquired and analyzed data. CYT and YCL assisted with the acquisition of funding and provided the research environment. YIC managed the execution of the experiments and animal models. SCL, YNC, and CCT have supervised and monitored the execution of this study. TCT and SLC made substantial contributions to the study concept, study design, and critical revision of the manuscript’s important intellectual content and submitted the manuscript for publication. All authors read and approved the final manuscript.

## Pre-publication history

The pre-publication history for this paper can be accessed here:

http://www.biomedcentral.com/1472-6882/14/30/prepub
